# Patient’s Perception About Coronary Artery Bypass Grafting

**DOI:** 10.5935/1678-9741.20150060

**Published:** 2015

**Authors:** Kelminda Maria Bulhões Mendonça, Tarcisio Matos de Andrade

**Affiliations:** 1 Hospital Universitário Professor Edgard Santos da Universidade Federal da Bahia (HUPES), Salvador, BA, Brazil.; 2 Faculdade de Medicina da Universidade Federal da Bahia (FMEB-UFBA), Salvador, BA, Brazil.

**Keywords:** Patients, Coronary Artery Bypass Grafting, Perception

## Abstract

**OBJECTIVE:**

The diagnosis of coronary artery disease referred for heart surgery has an important psychological component. The purpose of this study was to access the difficulties experienced by individuals awaiting coronary artery bypass grafting and to determine strategies that facilitate adaptation to a new lifestyle, modified by the disease.

**METHODS:**

A qualitative, exploratory study involving patients admitted to a university teaching hospital in the city of Salvador, Bahia, Brazil, awaiting coronary artery bypass grafting. Semi-structured interviews were performed in accordance with a previously defined script based on the study objective. Each transcription was read in its entirety to verify the representativeness, homogeneity and pertinence of the data obtained (pre-analysis), followed by separation of categories of analysis.

**RESULTS:**

The descriptions of this study show that patients admitted to the completion of coronary artery bypass grafting experience a wide range of psychological difficulties, considering that surgery acquires interpretations that vary according to individuals' subjectivity. The patients recognized the benefit of being able to discuss their feelings as a means of diminishing their fear and anxiety.

**CONCLUSION:**

Helping patients find resources to confront more positively the daily hospitalization is an important aspect for the health care professionals who assist them. This goal can be achieved through modification of the biomedical model of care for a biopsychosocial view. The investment of time and attention is of fundamental importance and aims to overcome existing deficiencies that interfere with the outcome of patients after cardiac surgery.

**Table:** 

**Abbreviations, acronyms & symbols**
CABG = Coronary artery bypass grafting

## INTRODUCTION

Coronary artery bypass grafting (CABG) is an important intervention for patients with coronary artery disease, bearing in mind that the procedure relieves angina and improves patients' quality of life. Nevertheless, while waiting to undergo CABG, patients experience distress, a sense of insecurity and altered quality of life^[1]^.

Cardiac surgery affects individuals in various ways. It is an invasive procedure in which the patient is susceptible to pain, infections and a risk of death. Insofar as the social sphere is concerned, the patients are separated from their friends and relatives, obliged to interrupt their professional life and, furthermore, their independence is restricted^[2]^.

Patients interpret cardiac surgery as an event associated with disablement and changes in body image. For many patients, it is a threatening procedure and inability to adapt to this situation results in increased anxiety^[3]^. These facts highlight a need to evaluate the patients' perception of the CABG procedure itself and the understanding of the possible effects of this perception on their recovery.

There is little detailed information in the literature on the perception of patients awaiting CABG^[4]^. Information on how patients experience this situation is important in order to enable those at risk of adverse psychological events to be identified. Based on the problems identified, interventions can be implemented that may help vulnerable individuals minimize their stress levels, potentially resulting in a more favorable outcome.

## METHODS

This qualitative exploratory study was performed between June and September 2013 by performing semi-structured interviews with patients admitted to the cardiology ward of the Professor Edgard Santos Teaching Hospital, Federal University of Bahia for CABG (Appendix 1 and 2, Interview 1 and 2).

Due to the qualitative nature of the study, the sample size was defined in accordance with the criterion of data saturation, i.e. recruitment stopped when the data supplied by the new participants added little to the material already obtained, with no further significant contribution being made towards improving the theoretical reflections based on the data that were being collected^[5,6]^. The saturation criterion was obtained after nine interviews, seven of which were conducted with male patients and two with female patients. Patients who participated in this study were between 45 and 65 years. All had a diagnosis of coronary artery disease and were awaiting CABG.

Patients scheduled to undergo cardiac surgeries other than CABG were not included in the study. Moreover, the inclusion criteria consisted of patients admitted to the cardiology ward prior to undergo CABG, who agreed to talk to the investigators about their expectations regarding the surgical procedure. The exclusion criterion was the presence of hemodynamic instability. None of the patients interviewed were using psychotropic drugs.

The interviews were conducted in a private setting and all of them were recorded, with the mean duration of each interview being 60 minutes. One of the interviewers listened to each recording within 48 hours of the interview to check that all the topics had been considered and to evaluate whether any additional information should be obtained. The quality and understanding of the content were also evaluated. The recordings were transcribed using the IBM VIAVOICE Pro USB software program.

Each transcription was checked for the representativeness, homogeneity and relevance of the data (a preliminary analysis). Next, the material was separated into categories of analysis; the content pertinent to each category was identified in each interview; and, finally, the results were treated (the actual analysis).

All the interviews were analyzed using discourse analysis, based on the thematic analysis, as proposed by Minayo^[5]^. Discourse analysis^[7,8]^ provides tools to analyze speech as an action and to examine how this action may reflect a given situation. The aim of the method of discourse analysis is not only to understand a message, but also to recognize its underlying meaning, i.e. its value and its dependence on a certain context.

## RESULTS

Data saturation was obtained after nine interviews, seven of which were conducted with male patients and two with female patients. The patients were between 45 and 65 years of age. All had been diagnosed with coronary artery disease and were awaiting CABG.

In the process of becoming ill, there is confrontation between the individual's life projects and his/her new reality. The patient begins to be treated as a function of the set of symptoms that he/she presents and not as a function of his/her singularity as an individual. Being admitted to hospital increases the sensation that the patient's routine has been disrupted and his or her autonomy lost, in certain circumstances leading the patient to review his/her life.

Some of the results found in the present study merit special attention as they highlight questions particularly related to the patients' experiences and expectations. The relevant data from each interview were allocated into blocks of content in accordance with the categories of analysis and illustrated with quotes taken from the patients' statements.

### Perceptions on life changes

The individuals interviewed expressed ambivalent feelings in relation to their indication for cardiac surgery. While recognizing that the intervention represented a means of improving their quality of life, they feared an unfavorable outcome. The following excerpts from their statements reflect what cardiac surgery meant to these patients:

"The surgery messes up my routine, but, before, I was unable to do anything and after the surgery I will be able to".

"I am nervous and this waiting period is very difficult. I heard that one patient died while waiting for surgery".

The difficulties were so great that a crisis was generated, temporarily disrupting the personality of some of the patients. While they waited for cardiac surgery, the patients felt deprived of their place of speech to talk about themselves:

"For me, time appears an eternity. I feel like an ET. People go back and forth, discuss my exam results and I'm just here looking on".

"When I went off to have an imaging exam, the doctor talked to a colleague about my results, but nothing was said to me".

It is obvious from the patients' speeches that in the process of becoming ill there is a confrontation between what the patient had envisaged for his/her life and the reality that is now presented to him/her in terms of their actual existence:

"What makes me apprehensive is this situation. I can't get this out of my head".

"It's as if you were a prisoner in the hospital. I want to get out of here and get back to living".

### Anxiety/Fear

Anxiety is one of the most common psychological diagnoses in the period preceding surgery. Cardiac surgery is a life-threatening experience for the patient and brings a significant emotional burden with it.

The patients interviewed in the present study spoke about their feelings of fear and discomfort triggered by the fact that they were waiting for cardiac surgery. The lack of perspective in relation to the future was described by some patients as the principal cause of these manifestations.

"...I keep imagining how things will be. I don't know what to expect, what will happen, how I will deal with this".

Explicitly or latently, fear was present in the statements of the patients faced with the perspective of having to undergo cardiac surgery. The following segments exemplify this feeling:

"The first thing you think is that something is going to go wrong during heart surgery".

"No one has explained what will happen during surgery; I'm waiting..."

In the present study, the patients experienced feelings of apprehension and helplessness. Some of the patients said that they had no opportunity to meet with the surgeon prior to surgery and had not therefore been able to obtain information on how the surgery would be:

"I know that I am going to have an operation, but I do not know how it is going to be; who is going to perform the surgery; how long it takes. I would like to have this information; it's important for me to meet the surgeon".

In addition, the patients had no information on the postsurgical period and rehabilitation. It is apparent from these reports that the patients have little information about the procedure to which they are to be submitted:

*"I would like the doctor to explain to me how my life will be after the surgery and if the doctor who will be operating on me will continue to follow me up"*.

### Denial

As a defense mechanism, denial allows the individual to remain psychologically intact by not accepting the problem. The intensity of this sentiment reflects the dimension of the impact of becoming ill in the patient's life.

The diagnosis of coronary artery disease has repercussions that are related to the symbolic representation of the heart, which led the individuals in the present study to a status of great vulnerability. The following statements show that the indication for cardiac surgery brings an expectation of rupturing the integrality of a vital organ, hence the patient's natural denial of his/her feelings and the need for the procedure.

"I'm not afraid. Perhaps I fear that after the surgery things will not be as expected...".

"My health is not good. I hope everything goes well. Sometimes I think I don't need surgery...".

### Concern with the family

A situation is evaluated as stressful when the individual identifies it as being life-threatening. The patients reported that during their hospital stay they experienced uncertainties and apprehension in response to the need to adapt to various changes in their routine.

"The problem is the expectation in relation to my family. They depend on me".

"I still don't have the information I need about how to control my anxiety. The symptoms get worse when I am stressed. I keep thinking about how my life and that of my family will be...".

The patients' perception of cardiac surgery was a factor that played a highly important role in defining the magnitude of their subsequent psychological symptoms. To protect themselves in this situation, the patients tried to think of other things in an attempt to reduce their feelings of distress caused by having to wait for cardiac surgery:

"When my brain is busy with work, I even manage to forget that I'm sick. It's important to keep my mind off the problem, even if just for a few minutes...".

The patients sought explanations for the situation they were experiencing. Some considered surgery a punishment for inappropriate behavior, thus assuming responsibility for the circumstances in which they now found themselves.

"I believe this is punishment. I have to face this realistically".

"There is a reason and an explanation for everything; nothing happens by chance. We are not entirely in charge of our destiny".

### The anesthesia

Anesthesia was an element that evoked conflicting feelings. Although anesthesia is given to avoid pain, to some of the patients it represented a complete loss of control over their own bodies, as shown in the following excerpt from a transcribed statement:

"When under anesthesia, I'll be asleep. I believe something could go wrong".

Since the heart is an organ that is imbued with symbolism, expectations in relation to anesthesia generate uncertainties, marking patients' subjectivity. The patients reported that their expectations in relation to anesthesia triggered distress at the thought that another individual would be responsible for their life at that moment.

"People forget that it is not a heart that is being operated on, but a person who has a heart. I am afraid of waking up during the surgery".

## DISCUSSION

Taking care of the patient in his/her integrality, with emphasis on the individual rather than on the disease, is a prerequisite for good quality healthcare. A diagnosis of coronary artery disease with an indication for surgery involves considering the patient's psychic component and it is imperative that the healthcare team is aware of this, particularly during the hospitalization period.

Surgery acts as a trial that enable the patients to take possession of the clinical condition they are experiencing. Surgery brought a burden of subjectivity to the individuals submitted to it and triggered fears that placed their lives on the edge.

The findings of the present study show that the patients who were awaiting CABG experienced a wide spectrum of psychological difficulties. The patients considered the diagnosis of heart disease and the waiting period prior to CABG as factors that negatively affected their expectations with respect to their work, routine and self-image.

The indication for CABG is particularly disturbing, since the heart is culturally regarded as the central organ of the body, the source of life and of the emotions^[9]^. As the time for surgery draws closer, the patients' emotional reactions intensify, as shown in their behavior, symptoms and, when given the opportunity, in words.

For these patients, surgery meant the end of a long period of having to live with deteriorating health and the beginning of a new life expectancy. Nevertheless, while the patient was hopeful in relation to the future, the surgery itself had to be dealt with. Despite the risks, the procedure represented an opportunity for survival.

The majority of patients with an indication for CABG report that fear, anxiety and uncertainty with respect to the future are more distressing than the chest pain^[10-12]^. Anxiety prior to cardiac surgery is a predictor of adverse events following surgery. Many patients fail to adapt and do not achieve the expected outcome, even when the surgical procedure is favorable^[13]^.

Irrespective of the individual distinctions in the reports included in this study, one characteristic common to all is the experience of a substantial amount of distress. As a result of this finding, the importance of reflecting on aspects of the cardiac surgery that go beyond the physical illness itself should be emphasized.

The patient's psychosocial status has a great influence on the results of the treatment of any disease, particularly when the aim of treatment is to improve quality of life. It is crucial to evaluate the patient's perception with respect to the stress generated prior to the surgical procedure and to understand the possible effects of this stress on the patient's physical and mental recovery^[14]^. The identification of predictive factors may improve the results of interventions for those at risk^[15]^.

Through the interviews conducted in the present study, it is clear that cardiac surgery involves feelings that the individual's integrity has been violated, and these feelings generate expectations with regard to the outcome. The interpretation of surgery should not be limited to a physical intervention, as it also involves significant changes in the way the patients see themselves and how they see others.

A high level of anxiety prior to cardiac surgery is a predictor of recurrence of the ischemic symptoms, of a greater number of re-admissions to hospital and a high degree of psychological stress following surgery^[16]^. The objectives of interventions designed to prepare patients for surgery include decreasing anxiety and fear by providing information and psychological support^[17]^.

The perspective of the patients and physicians in relation to cardiac surgery is affected by the associated existential conditions. The decision-making process within the doctor-patient relationship needs to be understood better and implemented in order to offer an appropriate approach for initiating a discussion on these issues^[18,19]^.

Healthcare should effectively provide the patient with integrated care, and this demands not only technical support but principally sensitive professionals who value the subjective aspects of the patients' lives, listen to their complaints and, together with the patients, identify strategies that will facilitate their adaptation to a new lifestyle, one that has been modified by the disease.

Recognizing the presence of defense mechanisms used by the patient in an attempt to adapt to the indication for CABG is fundamental in enabling interventions to be implemented to ensure a more favorable outcome. It is crucial that the multidisciplinary team be aware of the patients' demands in order to prepare them to get through the hospitalization period as tranquilly as possible.

The period preceding cardiac surgery demands much more from the healthcare professionals than just the preparations for the surgery itself, mainly with respect to the patient's psychological health. This care depends on a qualified individual listening to the patient and helping the patient feel secure, as well as on developing strategies to enable the patient to get through the hospitalization period as positively as possible.

## CONCLUSION

The aim of the present study was to emphasize the significant emotional burden that waiting for the surgical intervention triggers in these patients awaiting CABG. The form in which each individual confronts this intervention may facilitate his/her complete recovery and re-adaptation to normal life or hamper them.

Surgery acquires interpretations that vary in accordance with the patients' subjectivity, considering the change from the biomedical model of care to a biopsychosocial model. The data obtained at the interviews show that identifying how the patients deal with having to wait for cardiac surgery is of crucial importance for the multidisciplinary healthcare team, with the objective being developing interventions aimed at overcoming the deficiencies that currently affect the outcome of these patients.

The importance of reflecting on the aspects of cardiac surgery that go beyond the physical illness needs to be highlighted. An interdisciplinary team including a wide range of professional areas and knowledge should emphasize and evaluate these issues with the objective of comforting the patients waiting for CABG.

**Table:** 

**Authors' roles & responsibilities**			
KMBM	Analysis/interpretation of data; final approval of the manuscript; study design; implementation of projects/experiments; manuscript writing/critical review of its content		
TMA	Analysis/interpretation of data; final approval of the manuscript; study design; implementation of projects/experiments; manuscript writing or critical review of its content		
